# The impact of partial smokefree legislation on health inequalities: Evidence from a survey of 1150 pubs in North West England

**DOI:** 10.1186/1471-2458-5-91

**Published:** 2005-09-01

**Authors:** Karen Tocque, Richard Edwards, Brenda Fullard

**Affiliations:** 1 North West Public Health Observatory, Centre for Public Health, Liverpool John Moores University, North Street, Liverpool, L3 2AY, UK; 2 Evidence for Population Health Unit, Division of Epidemiology and Health Sciences, Stopford Building, University of Manchester, Oxford Road, Manchester, M13 9PT, UK; 3 South Sefton Primary Care Trust, 3 rd Floor Burlington House, Crosby Road North, Waterloo, Liverpool L22 0QB, UK

## Abstract

**Background:**

The UK government claims that between 10 and 30% of pubs and bars will be exempt from proposed legislation to achieve smokefree enclosed public places across England. This arises from the contentious inclusion that pubs and bars that do not prepare and serve food and private members clubs, will be able to allow smoking. We aimed to survey pubs and bars across the North West of England to assess smoking policies and the proportion and variations by deprivation level of venues preparing and serving food.

**Methods:**

We carried out a telephone survey of 1150 pubs and bars in 14 local authorities across the North West of England. The main data items were current smoking policy, food preparation and serving status, and intention to change food serving and smoking status in the event of implementation of the proposed English partial smokefree legislation.

**Results:**

29 pubs and bars (2.5%) were totally smoke-free, 500 (44%) had partial smoking restrictions, and 615 (54%) allowed smoking throughout. Venues situated in the most deprived quintiles (4 and 5) of deprivation were more likely to allow unrestricted smoking (62% vs 33% for venues in quintiles 1 and 2). The proportion of pubs and bars not preparing and serving food on the premises was 44% (95% CI 42 to 46%), and ranged from 21% in pubs and bars in deprivation quintile 1 to 63% in quintile 5.

**Conclusion:**

The proportion of pubs and bars which do not serve food was far higher than the 10–30% suggested by the UK government. The proportion of pubs allowing unrestricted smoking and of non-food venues was higher in more disadvantaged areas, suggesting that the proposed UK government policy of exempting pubs in England which do not serve food from smokefree legislation will exacerbate inequalities in smoking and health.

## Background

The English Public Health White Paper 'Choosing Health' and the accompanying delivery plan [1,2] state that the government will regulate, with legislation where necessary, to achieve smokefree enclosed public places and workplaces by 2007–2008. However, pubs and bars, which do not prepare and serve food and private members clubs, will be able to allow smoking. The UK government claim exempted pubs and bars represent between 10 and 30% of the total [1].

The Choosing Health proposals raise several concerns. One is that the non-smoking exclusion zone around the bar will be ineffective and harm to the health of staff working in pubs and bars and members clubs will continue. A second is that the effect of smokefree ordinances at reducing smoking prevalence [3] will be undermined by allowing smoking to continue in key social settings like pubs, clubs and bars. Finally, pubs and bars in disadvantaged areas may be less likely to serve food and, because of a higher smoking prevalence among customers, be most likely to stop serving food to allow continued smoking. This may undermine efforts to reduce smoking prevalence in these communities, and perpetuate and exacerbate the already gross inequalities in smoking [4,5] and smoking-related ill health.

Preliminary data from a survey of 29 Local Authorities conducted by the British Medical Association suggest that the 10–30% figure for exempted premises is an underestimate, particularly outside of the south of England. Thirteen out of 29 (10 located in the north or Midlands) councils estimated that more than 30% of their pubs did not serve food [6]. Figures from monitoring of pubs by Environmental Health Officers in Northamptonshire found that 54% of pubs and bars would be exempt, 85% in Corby, the most deprived Local Authority [7]. Similarly, a recent study of the catering status of 174 pubs on the Local Authority records of Telford and Wrekin borough found that 43% of pubs would be exempt, 69% in the most deprived areas [8].

We conducted a survey across 14 Local Authorities in the North West region to assess current smoking policies, the proportion of pubs and bars preparing and serving food, and variations in this proportion by deprivation level of the local area. We hypothesised that pubs and bars from disadvantaged areas would be more likely to allow unrestricted smoking and less likely to prepare and serve food.

## Methods

A structured questionnaire (available online with methodological instructions [9]) was adapted from a pilot survey carried out in Wirral Local Authority and disseminated to all 43 Local Authorities within the North West region. Fourteen Local Authorities agreed to participate: six were predominantly urban, three were predominantly rural and five were mixed urban/rural. Within each Local Authority, a local coordinator identified lists of licensed premises. Either all or a random sample of licensed premises were chosen for inclusion. At least three attempts were made to make contact with the proprietor or owner of chosen establishments. A telephone interview was conducted using the questionnaire except in a few cases where the questionnaire was completed in a face-to-face interview.

Some areas focused only on pubs, bars and clubs, whereas others also included hotel and restaurants with licensed bars. The current analysis is largely restricted to pubs and bars and private members clubs, and excludes night clubs, restaurants and hotels. Venues were allocated an area-based deprivation score (Index of Multiple Deprivation (IMD) 2004 [10]) by mapping their postcode to Census Super Output Area which were categorised into deprivation quintiles for all Super Output Areas across the north west.

## Results

There were 1818 licensed pubs, bars included in the sampling frame, of which 1150 pubs and bars (63%) agreed to take part, 386 (21%) refused, and 282 (16%) could not be contacted. Of 396 members clubs contacted 195 (49%) responded and 78 (20%) refused and 123 (31%) could not be contacted. The interviewee was the manager or proprietor for 73% of pubs and bars participating.

Across the 14 Local Authorities, only 29 pubs and bars (2.5%) were totally smoke-free, 500 (44%) had partial smoking restrictions, and 615 (54%) allowed smoking throughout. Seventy-one per cent allowed smoking at the bar.

The proportion of pubs and bars not preparing and serving food on the premises was 44% (95% Confidence Interval (CI) 42 to 46%), and ranged from 19 to 55% across the 13 Local Authorities (one Local Authority excluded because only 14 venues participated). Respondents were asked if they were likely to change their policy on preparing and serving food in response to the English Public Health White Paper proposals. Of pubs and bars that currently served food, 82 (13%) stated that they were likely to stop serving food, and of those that didn't serve food 42 (9%) stated that they were likely to start serving food in response to the proposed English smokefree legislation. The net projected change in the number of venues that would prepare and serve food after introduction of the White Paper proposals was a reduction of 40 (3%), increasing the proportion of non-food pubs from 44 to 47%.

**Figure 1:**
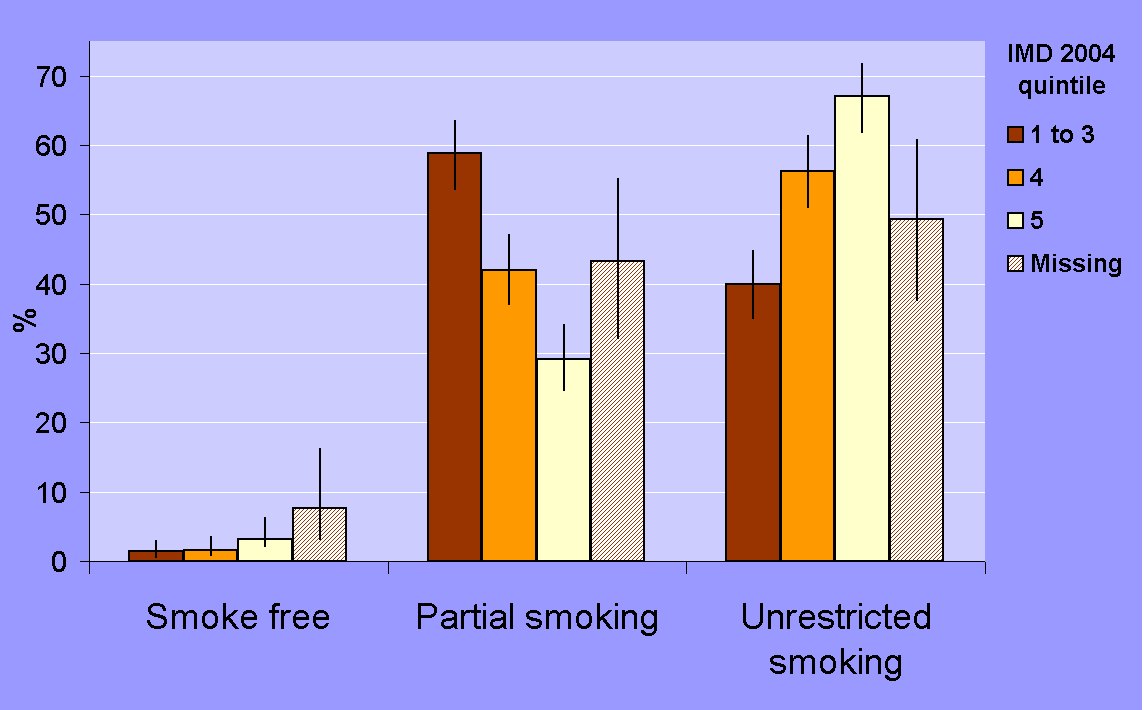
**Current smoking policies in North West pubs and bars by deprivation quintile (IMD 2004). Data showing percent of venues with 95% confidence intervals**. IMD quintile was allocated by the Census Super Output Area (SOA) of the venue as determined by the postcode of the venue. Quintile 1 represents the most affluent 20% of SOAs and quintile 5 represents the most deprived 20% of SOAs in the North West region.

Results stratified by IMD-2004 quintiles are shown in table 1. The proportion of venues with unrestricted smoking increased with deprivation (Figure 1). In the more affluent areas only 21% (IMD-2004 quintile 1) and 40% (quintile 2) allowed smoking throughout compared with 56% in deprivation quintile 4 and 67% in the most deprived quintile 5 – the absolute difference in proportions between venues in quintiles 1–2 and quintile 4–5 was 28%, (95% CI 21 to 36%).

**Table 1:** Smoking and food preparation policies by IMD-2004 quintiles of deprivation in pubs and bars in 14 Local Authorities in the North West of England.

	All (n = 1150)	IMD-2004 Quintile					IMD-2004 Missing (n = 70)
			
		1 (n = 70)	2 (n = 126)	3 (n = 180)	4 (n = 355)	5 (n = 349)	
% with unrestricted smoking	53.8	21.4	39.7	47.2	56.3	67.1	49.3
% allowing smoking at the bar	70.7	55.7	66.7	68.3	71.8	77.9	57.1
% currently not preparing and serving food	43.6	21.4	28.6	38.9	36.9	63.3	40.0
% not preparing and serving food post White Paper (predicted)	47.0	20.0	31.8	41.1	46.2	62.8	41.4
% of food pubs allowing smoking in food serving areas	75.7 (n = 649)	80.0 (n = 55)	74.4 (n = 90)	76.4 (n = 110)	75.0 (n = 224)	67.2 (n = 128)	64.3 (n = 42)
% of pubs allowing children on premises	60.3	77.1	79.4	69.4	52.1	54.2	57.1
% of pubs allowing children on premises with smoking in children's areas	81.4 (n = 693)	85.2 (n = 54)	83.0 (n = 100)	81.6 (n = 125)	75.7 (n = 185)	78.3 (n = 189)	75.0 (n = 40)

The Index of Multiple Deprivation 2004 (IMD 2004) is a Super Output Area (SOA) level measure of multiple deprivation and is made up of seven SOA level Domain Indices: Income deprivation, Employment deprivation, Health deprivation and disability, Education, skills and training deprivation, Barriers to Housing and Services, Living environment deprivation and Crime. The overall IMD is conceptualised as a weighted area level aggregation of these specific dimensions of deprivation [12].

**Figure 2:**
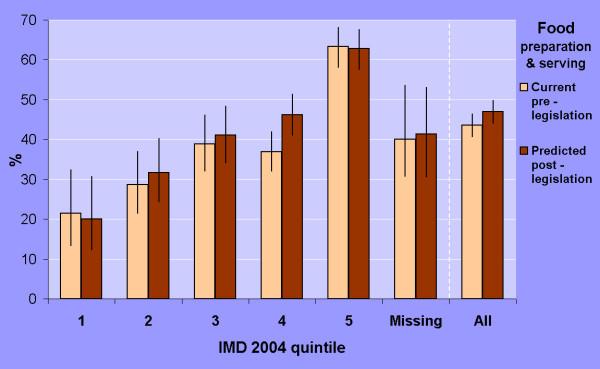
**Current and predicted preparation and serving of food in North West pubs and bars by deprivation quintile (IMD 2004). Data showing percent of venues with 95% confidence intervals**. IMD quintile was allocated by the Census Super Output Area (SOA) of the venue as determined by the postcode of the venue. Quintile 1 represents the most affluent 20% of SOAs and quintile 5 represents the most deprived 20% of SOAs in the North West region.

The proportion of pubs and bars not preparing and serving food increased with the level of deprivation (Figure 2) from: 21% in the most affluent areas (IMD-2004 quintile 1) to 37–39% in quintiles 3 and 4, and 63% in quintile 5 (χ^2 ^= 65.1, p < 0.001). Difference in proportions between quintiles 1 and 5 was 42% (95% CI 30% to 51%). Based on respondents' stated intentions, the English Public Health White Paper proposals are likely to exaggerate further the gradient in the proportion of non-food pubs and bars by deprivation level (Figure 2).

Over half of the venues allowed children on the premises. This proportion was lowest in venues in the most disadvantaged areas. Around 80% of pubs and bars where children were allowed permitted smoking in the children's areas. This varied little by IMD-2004 quintile (Table 1). Over three quarters of pubs which prepared and served food allowed smoking in the areas where food was served. This was slightly less in venues in the most deprived quintiles.

Of licensed members clubs, 6% were smokefree, 33% had partial smoking restrictions and 61% allowed unrestricted smoking. There was a gradient in the proportion allowing unrestricted smoking, from 31% in IMD-2004 quintile 1 to 68% in quintile 5.

## Discussion

We found that 44% of pubs and bars across 14 Local Authorities in the North West of England do not prepare and serve food – far higher than the 10–30% claimed by the UK government [1]. Furthermore, more respondents indicated that they would stop rather than start serving food in response to the White Paper proposals, resulting in a projected increase of 3% in the proportion not preparing and serving food.

There was a strong socio-economic gradient in the distribution of non-food serving pubs and bars: with 63% of businesses located in the most deprived areas (quintiles 4 and 5) not preparing and serving food. Unrestricted smoking was also much more likely in pubs, bars and members clubs in the most deprived areas.

This is the largest and most comprehensive survey of the likely impact of the English smokefree proposals from over 1100 pubs and bars from a wide range of Local Authorities across the North West. It explores variations by level of deprivation below Local Authority level of aggregation and is the first to examine likely changes to food serving policy after the proposed legislation is implemented. The findings are based on data collected directly from pubs and bars rather than estimates made by Local Authorities [6].

The White Paper Smokefree Consultation document [11] notes that it has been suggested that the 'Choosing Health' proposals will result in smoking pubs and bars being concentrated in deprived communities, thereby exacerbating health inequalities. This is confirmed by our survey, which found that pubs and bars that don't serve food and hence will be able to allow smoking after 2008 are more concentrated in disadvantaged areas in the North West. It is highly probable that this socio-economic gradient in the location of food/non-food serving establishments will also exist in other parts of England.

## Conclusion

The impact of the 'Choosing Health' proposals are likely to contribute to maintaining the huge inequalities in smoking prevalence and smoking-related morbidity and mortality by perpetuating a strong smoking culture, reducing the impact of cessation in response to smokefree policies, and maximizing exposure of bar staff and non-smoking customers to passive smoking in the most deprived areas. All these work against the stated Government objective of reducing health inequalities due to smoking.

## Competing interests

The author(s) declare that they have no competing interests. Richard Edwards is unpaid chair of North West ASH and Brenda Fullard is seconded to the North West Public Health Team, Government Office for the North West.

## Authors' contributions

Following a pilot survey carried out by Tina Williams at Smokefree Wirral, KT, RE and BF developed the idea for a regional survey and modified the questionnaire. KT designed the data collection system, co-ordinated the distribution of the questionnaire and collation of the data. Data collection was organised by several local co-ordinators. KT and RE planned and carried out the analysis. The paper was drafted initially by RE, and then developed further with contributions from all the co-authors.

## Pre-publication history

The pre-publication history for this paper can be accessed here:


